# Endoscopic endonasal surgery for giant pediatric craniopharyngioma

**DOI:** 10.3171/2020.4.FocusVid.19983

**Published:** 2020-04-01

**Authors:** Ahmed Mohyeldin, Peter Hwang, Gerald A. Grant, Juan C. Fernandez-Miranda

**Affiliations:** Departments of 1 Neurosurgery and; 2 Otolaryngology, Stanford University Medical Center, Palo Alto; and; 3 Pediatric Neurosurgery, Lucile Packard Children’s Hospital, Stanford, Palo Alto, California

**Keywords:** pediatric brain tumors, craniopharyngioma, skull base, endoscopic endonasal surgery, surgical video

## Abstract

Pediatric craniopharyngiomas that were once thought to be inoperable or considered only for salvage medical therapy are now being reconsidered for aggressive surgical resection via endoscopic endonasal approaches. Here we review the operative video case of an 11-year-old with a giant complex craniopharyngioma that was resected via an endoscopic endonasal approach. Due to the extent of tumor burden near the basilar apex, a transclival approach was necessary. To accomplish this, a wide sphenoidotomy, posterior ethmoidectomy, and resection of the middle turbinate were necessary to create enough working space for the resection. We also highlight several key innovations in pediatric endoscopic endonasal surgery management and underscore a multidisciplinary approach that allows for the safe and successful treatment of these lesions. Our multidisciplinary team involves an experienced fellowship-trained endoscopic skull base surgeon and otolaryngologist, as well as a pediatric neurosurgeon, pediatric endocrinologist, pediatric anesthesiologist, and pediatric intensivists who play important roles in the preoperative, intraoperative, and postoperative phases of care of the patient. Finally, we discuss critical surgical decision points including pituitary transposition, which has a lot of conceptual appeal when it is anatomically feasible but unfortunately, in our experience, has low functional preservation rates. Initially, we always aim to utilize pituitary transposition for tuberoinfundibular craniopharyngiomas, and once the relationship between the tumor and the stalk is determined, a decision on whether to preserve or sacrifice the stalk and pituitary gland is made. In this particular case, there was a salvageable stalk and the transposition was performed knowing that the chances for functional preservation were low.

The video can be found here: https://youtu.be/ClL73FU5QIU.

## Transcript

This is Dr. J.F.M. presenting a giant pediatric craniopharyngioma case, one of the most formidable intracranial tumors we can do as surgeons. This is an 11-year-old male patient that had an Omaya reservoir placed. In spite of that, he has developed multiple cysts. A large calcified mass compatible with a craniopharyngioma. This is the virtual reality simulation using a fine-cut CT angiogram to identify the relationship of the tumor, in particular the calcified portion of the tumor with the surrounding neurovascular structures. We are now looking at anatomy from the front simulating an endonasal endoscopic approach. We can see the sphenoid sinus and the tumor located above and behind the sella between the pituitary gland. We can also identify the relationship of the tumor with the neurovascular structures, the arteries of the circle of Willis. We can see the carotid artery bifurcation. The basilar bifurcation with the posterior communicating arteries and the posterior cerebral arteries intimately involved with the tumor.

**1:43** We are now in the operation.

**1:45** We started the drilling, identifying the optic nerve prominences, the carotid prominences in the clinoidal space. Of note, as it happens frequently in pediatric skull base cases, the sphenoid sinus is poorly pneumatized. So the first thing we need to do is to recreate the anatomy of the sphenoid sinus by recreating the clival recess below the sella. We’re taking pieces of the floor of the sphenoid sinus, and we are now transecting the pharyngobasilar fascia in the midline, and this facilitates further, lower clival drilling.

**2:24** Now we’re doing it by the optic carotid confluence, removing the bone at the level of the tuberculum sella first. Now removing the bone that covers the anterior wall of the cavernous sinus on the right side. Complete removal of the tuberculum sella bone. And now this is bone being removed to uncover the clinoidal carotid artery on the left side of the patient.

**2:51** Further drilling allows us to expose the anterior wall of the cavernous sinus on the right side and trying to expose the clinoidal artery on the right side. But I find that there is a prominent medial clinoidal ring, which I left in place. Now I am removing the bone of the dorsum sella. Now extending the clivectomy inferiorly and finally, we were able to open the dura. We start at the sella. We coagulate the superior intercavernous sinus, and now I start by mobilizing the pituitary gland off the sella. I find the so-called pituitary ligaments, which can be transected easily, and I have to completely rotate the pituitary gland away from the sella. I identify a branch of the superior hypophyseal artery that is going to the gland that is coagulated and transected. I identify the inferior hypophyseal artery running through the floor of the sella. I coagulate it, and then I can transect the dura of the dorsum sella completely, and this allows complete mobilization of the pituitary gland, so-called full pituitary intradural transposition. Now I have opened the dura of clivus all the way down so I can identify intradurally the arachnoid membranes of the prepontine cistern. I can see the basilar artery. We can see the pons; we can see the perforating branches going to the pons.

**4:24** And now we enter into the cyst first and then into the calcified portion of the tumor. We identify the third nerve on the left side. We continue with progressive debulking. We find probably the anterior inferior cerebellar artery. First on the left, now on the right, and the right side, the branch of the anterior inferior cerebellar artery are already are quite attached to the tumor capsule. So this requires very careful dissection for the attachment and preservation of these vessels. Progressive debulking of the tumor with sharp dissection, two suctions technique, extracapsular dissection again allows me to identify the left posterior communicating artery. The third nerve that has been compressed, flattened by the tumor. I continue progressing now superiorly toward the retrosellar space following the dissection of the PComm on the left side and of the third nerve. We are now working between the PComm and the superior hypophyseal artery. A branch from the superior hypophyseal artery has to be transected because it goes directly into the tumor. We’re working above the PComm on the left side. This debulking allows for further mobilization and extracapsular dissection. Now I am working on the right side and I find the right oculomotor nerve.

**6:03** Capsule of the tumor is being been removed. I identify the superior cerebellar artery above the third nerve, and now I identify this very small posterior communicating artery and now directly dissecting at the basilar bifurcation and finding this plane of dissection between the vessels and the tumor, as a tumor goes and enters the third ventricular cavity. That is the cerebral peduncle, and then I am finding the subarachnoidal plane to nicely preserve the neurovascular structures while I mobilize the tumor away. Further debulking of the tumor exposes the ventricular catheter that had been placed before. That’s the catheter for the Omaya reservoir. I have to further mobilize pituitary gland to access the tumor behind it. I am using a right-angle knife to facilitate this dissection, and I’m able to remove the cyst which was extending very lateral to the medial temporal lobe, which can be seen laterally. Working now superiorly behind the pituitary gland toward the third ventricle. I start seeing the walls of the hypothalamus, which I’m protecting with a cottonoid. And we can see now that inside of the third ventricle. We do a very careful, sharp dissection of the tumor.

**7:42** The pituitary gland is being again mobilized at the same time and trying to identify the branches of the superior hypophyseal artery that should be preserved because they actually go to the optic apparatus and at the same time, we do continuous debulking of the tumor.

**7:58** And again dissecting this tumor off the hypothalamic wall, now on the left side. The endoscope and endonasal approach provide unique visualization of this interface between the tumor and the hypothalamus.

**8:17** I can directly identify the hypothalamus the inside of the third ventricle and I continue. I can continue the trimming of the tumor to a safe plane of the dissection. That is in the retrochiasmatic space. A touch of the hypothalamus and, again, we’re doing sharp dissection, which is the best way to prevent injury to the hypothalamus. That is the attachment and probably the origin of the tumor. I am now working superiorly above the pituitary gland in the retro- and the infrachiasmatic space detaching tumor from the optic apparatus, while at the same time preserving the small branches from the superior hypophyseal artery. We can see the PComm on the right side, that is, the pituitary gland, and now we’re looking into the third ventricle, foramen of Monro. We can see the left hypothalamus, which is the origin of this particular tumor, and this is at the end of the resection with the pituitary gland toward the right side, the basilar bifurcation, the optic chasm. All neurovascular structures preserved and a complete resection of this tumor. Reconstruction is now performed with an inlay layer of DuraGen, then fascia latta, then a fat graft to occupy the clival recess. And finally, a large extended nasal septal flap that perfectly covers all the defect. This is reinforced and secured with layers of surgical tissue glue and Gelfoam. An intraoperative MRI was completed showing complete resection of the tumor with an excellent reconstruction.

**10:16** And patient had an excellent outcome.
